# Factors influencing the relationship between cochlear health measures and speech recognition in cochlear implant users

**DOI:** 10.3389/fnint.2023.1125712

**Published:** 2023-05-12

**Authors:** Ladan Zamaninezhad, Berkutay Mert, Heval Benav, Jochen Tillein, Carolyn Garnham, Uwe Baumann

**Affiliations:** ^1^MED-EL GmbH, Innsbruck, Austria; ^2^ENT/Audiological Acoustics, University Hospital, Goethe University Frankfurt, Frankfurt, Germany

**Keywords:** cochlear implant, cochlear health, speech recognition, neural degeneration, band importance function, age

## Abstract

**Background:**

One factor which influences the speech intelligibility of cochlear implant (CI) users is the number and the extent of the functionality of spiral ganglion neurons (SGNs), referred to as “cochlear health.” To explain the interindividual variability in speech perception of CI users, a clinically applicable estimate of cochlear health could be insightful. The change in the slope of the electrically evoked compound action potentials (eCAP), amplitude growth function (AGF) as a response to increased interphase gap (IPG) (IPGE_*slope*_) has been introduced as a potential measure of cochlear health. Although this measure has been widely used in research, its relationship to other parameters requires further investigation.

**Methods:**

This study investigated the relationship between IPGE_*slope*_, demographics and speech intelligibility by (1) considering the relative importance of each frequency band to speech perception, and (2) investigating the effect of the stimulus polarity of the stimulating pulse. The eCAPs were measured in three different conditions: (1) Forward masking with anodic-leading (FMA) pulse, (2) Forward masking with cathodic-leading (FMC) pulse, and (3) with alternating polarity (AP). This allowed the investigation of the effect of polarity on the diagnosis of cochlear health. For an accurate investigation of the correlation between IPGE_*slope*_ and speech intelligibility, a weighting function was applied to the measured IPGE_*slopes*_ on each electrode in the array to consider the relative importance of each frequency band for speech perception. A weighted Pearson correlation analysis was also applied to compensate for the effect of missing data by giving higher weights to the ears with more successful IPGE_*slope*_ measurements.

**Results:**

A significant correlation was observed between IPGE_*slope*_ and speech perception in both quiet and noise for between-subject data especially when the relative importance of frequency bands was considered. A strong and significant correlation was also observed between IPGE_*slope*_ and age when stimulation was performed with cathodic-leading pulses but not for the anodic-leading pulse condition.

**Conclusion:**

Based on the outcome of this study it can be concluded that IPGE_*slope*_ has potential as a relevant clinical measure indicative of cochlear health and its relationship to speech intelligibility. The polarity of the stimulating pulse could influence the diagnostic potential of IPGE_*slope*_.

## 1. Introduction

Cochlear implants (CIs) are the treatment of choice to restore hearing in patients with severe to profound hearing loss (HL). The success of the treatment depends on individual factors such as the cognitive abilities of the patient or the reaction of the immune system to the implant, as well as on implant type and the depth of insertion of the electrode array. One influential factor is the condition of the cochlea –Specifically the survival and the physiological status of the spiral ganglion neurons (SGN). Although the importance of this factor is clear, relevant data are sparse.

Spiral ganglion neurons are the target neurons for electrical stimulation with cochlear implants. Large variations have been documented in the number and condition of surviving SGNs in CI recipients, and this could contribute to the similarly large variability in auditory performance observed (Seyyedi et al., 2014). The parameters describing the status of the auditory nerve include the number of SGNs (neural count), the presence, density, and myelination of their peripheral processes (PP), and metabolic and genetic factors. In this paper, we will use “cochlear health” as a generally inclusive term to encompass all of these parameters.

Some of the early attempts to correlate speech recognition in CI users with cochlear health used post-mortem histology of the temporal bone. These studies showed either negative (Nadol et al., 2001) or no correlation (Khan et al., 2005) between residual SGN counts and word recognition. Potential reasons for the lack of correlation include the long duration between speech recognition testing and histological analysis, the limited datasets, the use of pooled data across the electrode array, the use of only neural count, excluding condition, and the inter-individual differences in cognitive abilities. Seyyedi et al. (2014) presented the first study data which showed a positive correlation between the number of surviving SGNs and word recognition score. In a within-subject comparison of left and right ears, eliminating any between-subject confounding factors, the number of SGNs was consistently higher in the ear which produced the better word recognition scores. Despite the positive correlation observed between cochlear health and auditory performance in CI users, several of the limitations of previous studies were also present here. The study was also conducted post-mortem, the demonstrated differences were small, histological data were again pooled across the cochlea, and only very limited data were available.

Another aspect of cochlear health, genetic factors, can influence the function of the auditory nerve (Shearer et al., 2017; Shearer and Hansen, 2019; Usami and Nishio, 2022). Several attempts have been made to establish Cochlear Nerve Deficiency (CND) as a reference model for verifying cochlear health measures (He et al., 2018, 2020; Xu et al., 2020). However, these findings were not as expected, and some measures for cochlear health were found to be contrary to the initial hypothesis (Xu et al., 2020). This may be due to the pathogenesis of CND, in which the status of the auditory nerve is affected during embryogenesis (Jackler et al., 1987) and the frequent presence of concurrent neurological deficits (Huang et al., 2010). These findings may suggest that patients with CND may not be a suitable model for general investigation of cochlear health (Xu et al., 2020).

Electrically evoked compound action potentials (eCAPs) may provide a means for the estimation of cochlear health in live individuals. The eCAP represents the synchronous ensemble activity of electrically stimulated auditory nerve fibers, and has the same neural origin as Wave I of the electrically evoked auditory brainstem response (eABR). Its primary constituents are a negative (N1) peak, which occurs at approximately 0.2–0.4 ms after stimulus onset, followed by a positive (P2) peak at 0.6–0.8 ms. Several characteristics of the eCAP could potentially be examined to glean information regarding cochlear health (van Eijl et al., 2017). DeVries et al. (2016) observed a negative correlation between eCAP amplitude and behavioral thresholds, and reported a tendency toward better speech perception for subjects with higher eCAP amplitude and lower behavioral thresholds. Kim et al. (2010) observed a significant correlation between the slope of eCAP amplitude growth function (AGF) and speech recognition in quiet and in noise, although only for the sub-group of participants with short electrode arrays.

**FIGURE 1:**
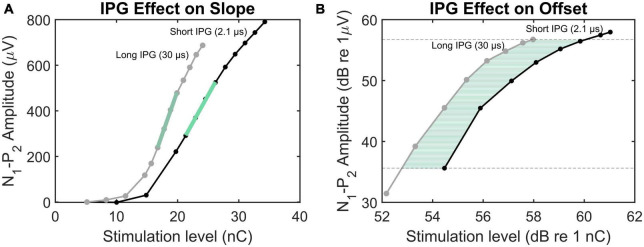
Illustration of the methods employed to calculate IPGE_*slope*_ **(A)** and IPGE_*offset*_ **(B)**. The eCAP AGFs obtained by IPG 2.1 and 30 μs are plotted in black and gray, respectively. In panel **(A)**, the green lines mark the AGFs steepest slopes. In panel **(B)**, the green horizontal lines indicate the offset between the AGFs with short and long IPGs for several N1-P2 amplitudes.

To mitigate the site-specific variation in eCAP response caused by non-neural factors such as the distance between each electrode and its target SGNs, as well as tissue or bone growth, Prado-Guitierrez et al. (2006) introduced a method based on alteration of the interphase gap (IPG, Figure 1A). The IPG is the short zero-amplitude portion between the anodic and cathodic phases of a symmetric, charge-balanced biphasic pulse used in CIs. The effect of increasing the IPG (IPG effect, IPGE) of the stimulating pulse on eCAP characteristics has become widely used as a measure of cochlear health. Ramekers et al. (2014) measured the IPGE on different eCAP AGF characteristics such as amplitude, threshold, slope, and latency in implanted normal-hearing (NH) and pharmaceutically deafened guinea pigs to investigate the consequences of secondary degeneration of SGNs after severe hair cell loss through chemical ablation. A significant correlation between spiral ganglion cell packing density and the IPGE on some of the AGF characteristics, including slope, was shown.

The same method was used by Schvartz-Leyzac and Pfingst (2016) for estimation of cochlear health in human subjects. The authors measured the IPGE on the AGF slope (IPGE_*slope*_) and observed across-site profiles reflecting the local variability along the cochlea. In a subsequent study (Schvartz-Leyzac and Pfingst, 2018), each ear was represented by the across site mean (ASM) of the measured IPGE_*slope*_ for all electrodes. Similarly to Seyyedi et al. (2014), by calculating the ear-difference in the IPGE_*slope*_ ASM in bilateral CI users, the between-subject bias stemming from variation in central cognitive ability could be reduced. A significant correlation between ear-differences in IPGE_*slope*_ and ear-differences in speech reception threshold (SRT) was shown. The approach proposed by Schvartz-Leyzac and Pfingst (2018) was challenged by Brochier et al. (2021) who investigated the IPG effect on the eCAP AGF in computational and animal models. Based on both models, they concluded that IPGE_*slope*_ did not successfully control for non-neural factors. Instead, they proposed the IPGE on offset (IPGE_*offset*_), defined as the average dB offset between the overlapping linear regions of the two eCAP AGFs obtained with a short and a long IPG (Figure 1B) that are expressed on logarithmic input-output axes. The contradictory conclusions of the two above-mentioned studies emphasized on the necessity of further research to clarify the suitability of each measure for estimation of cochlear health.

Speech information in different frequency bands, transmitted from different sections of the cochlea to the brain, is not of equal importance (ANSI, 1997). Because of this, two ears with the same ASM of the IPGE on eCAP characteristics may differ in speech perception if the distribution of surviving SGNs differs between each cochlea. Regardless of the type of cochlear health measure, when it comes to relating the measure to speech recognition performance, any such measure may benefit from an adjustment using a band importance function that reflects the human auditory system mechanisms of speech perception.

Between-subject variations in the site-specific cochlear capacity for transferring speech information has been considered for other measures related to speech intelligibility. The speech intelligibility index predicts the intelligibility of speech based on the sum of speech audibility in different frequency bands multiplied by the importance of each band, which is determined by that band’s contribution to the intelligibility of total speech information (ANSI, 1997). This function has been estimated in normal hearing subjects using recognition scores of successively low- and high-pass filtered speech. The importance of a band is then determined by comparing the recognition scores across two successive cutoff frequencies (Healy et al., 2013). The perception of the masked speech signal depends on the noise spectrum and differs through variations in sub-band signal-to-noise ratio (SNR) even for the same subject as reported by Pollack (1948) who measured the intelligibility of low- and high-pass filtered speech masked with white noise. The author reported a relative contribution of the various speech frequencies as a function of SNR. On the other hand, the speech perception of hearing impaired individuals with different patterns in their audiograms varies even under the same noise condition. In case of profound HL and in the absence of functional hair cells, variation in neural survival along Rosenthal’s canal influences the transmission of speech in each frequency band. We therefore conclude that it may be advisable to refine the method developed by Schvartz-Leyzac and Pfingst (2018) and to consider the speech band importance function when relating the IPGE to speech perception.

Another factor that might affect the diagnostic power of the IPGE is the polarity of the stimulating pulse. Histological studies have shown that SGN degeneration occurs over an extended period of time in the human and after loss of supporting cells and degeneration of SGN PPs can also survive as monopolar neurons (Liu et al., 2015; Wu et al., 2019). Models predict that SGNs with degenerated PPs require 5 to 6 times more current than healthy neurons to respond to cathodic-leading pulses, however, this is not true for anodic-leading pulses (Rattay et al., 2001a,b; Joshi et al., 2017; Resnick et al., 2018). Single-fiber recordings (Shepherd and Javel, 1997) and investigations of pseudo-monophasic and triphasic pulses (Undurraga et al., 2010, 2013) have provided electrophysiological evidence for this hypothesis. Biophysical considerations on the effect of externally applied electrical fields on neuronal excitation assign an important role to the polarity of the stimulus (Kalkman et al., 2022). Here, a significant factor is the orientation of the neuron in relation to the orientation of the voltage gradient caused by the electric field. In general, cathodic stimulation is seen as more effective for neuronal stimulation (Rattay, 1998). The electrode generates negative potentials in the extracellular space, such that the intracellular potential is no longer negative (Brocker and Grill, 2013) and the transmembrane potential is depolarized, leading to an action potential. This is, however, dependent on the distance of the electrode to the target neuron, which in the case of CI stimulation never are in close contact and are separated by perilymph as well as multiple tissues. In an analogous way, anodic stimulation can create “virtual cathodes” in locations distant from the electrode. CIs often evoke larger eCAPs with anodic stimulation (Macherey et al., 2008; Herrmann et al., 2021). Degeneration and demyelination of the SGN peripheral process effectively moves the neuron further away from the electrode, compared to a healthy SGN (Kamakura et al., 2018). Factors increasing electrode-neuron distance may be creating a preference for anodic stimulation by an electrode. The “generalized activating function” (Rattay, 1999) observes spatial and temporal local voltage changes, which depend on extracellular voltages, axonal resistance, and membrane capacitance. On a single-cell level, the simulations showed that the amount of threshold increase following the loss of the peripheral processes essentially depended on the electrode position and the polarity of the stimulus. Degeneration and demyelination of PPs is predicted by all these models to lead to more significant loss of efficiency for cathodic stimuli, due to more suitable alignment of excitable regions of the SGN and excitatory extracellularly applied potentials in the anodic case. However, often both anodic-leading and cathodic-leading stimuli are used in a single eCAP measurement and then averaged for purposes of artifact reduction (alternating polarity, AP), making a differentiated observation for single polarities impossible. The model outcomes motivated a comparison of the diagnostic potential of anodic-leading stimuli versus that of cathodic- leading stimuli alone for objective assessment of cochlear health. The forward masking (FM) artifact reduction method uses two biphasic pulses with the same leading phase polarity, most commonly a cathodic-leading pulse (He et al., 2017). It is also possible to implement FM with an anodic-leading pulse. The hypothesis of this study is not directed at the polarity effect on excitability as investigated in previous studies (Hughes et al., 2017, 2018; Jahn and Arenberg, 2019a,b), which focused on the average differences of the responses to anodic-leading and cathodic-leading stimuli. Instead, both polarities are investigated separately in the current study to examine the sensitivity of each polarity separately for use as an electrophysiological biomarker for SGN degeneration.

In summary, the goals of this study were to investigate the relationship between IPGE_*slope*_, demographics, and speech intelligibility in CI users by (1) by investigating the effect of the polarity of the stimulating pulse, and (2) considering the band importance weighting function when investigating the correlation between the speech perception measures and IPGE_*slope*_ within the study.

## 2. Subjects and methods

### 2.1. Subjects

The subject cohort consisted of 13 bilateral CI users with a mean age of 58 years (range 29–91). Detailed demographic data are given in Table 1. We recruited CI users implanted with MED-EL devices to ensure compatibility with the custom-made eCAP measurement software described below. Etiology varied between subjects, with the most common etiology being progressive sensorineural HL (10 subjects, at least in one ear). All subjects were native German speakers and were stimulated in monopolar mode. For all the subjects the FS4 coding strategy was used and the lowest frequency was set to 70 Hz. Individuals whose HL was secondary to meningitis were excluded from this study, due to the reduced incidence of recordable eCAPs in this condition (Guedes et al., 2007). Subjects 7, 9, 12, and 13 suffered from progressive hearing impairment which was detected prelingually. Subjects 7 and 13 had restricted speech development and were diagnosed with mild auditory dyslalia. Electrode 12 of the right ear of subject 12 and electrodes 4 and 5 of the left ear of subject 7 were deactivated clinically. In the left ear of subject seven, the IPGE_*slope*_ measurement of electrodes 3, 7, and 10 was interrupted due to the subject’s complaint of an unpleasant sensation. The left ear of subject seven was the only ear implanted with a relatively short electrode array (Flex24 EAS). Recruitment of subjects for this study was approved by the Ethics Committee of the Goethe University Hospital in Frankfurt (ERB number 44/19), and all subjects gave written informed consent. Subjects received an expense allowance for participation in the study.

**TABLE 1:** Demographic data and summary statistics are provided for age, duration of hearing loss until CI-implantation, hearing aid experience, CI experience and residual hearing.

ID	Age	Duration of HL until Cl-		Δ duration of HL	Hearing aid experience		ΔHA experience	Cl experience		ΔCl experience	PTA-LOW (dB HL)		Sex	Etiology	Implant		Electrode		Processor	
		L	R		L	R		L	R		L	R			L	R	L	R	L	R
Sbj 2	45	37.6	37.6	0	27.96	27.96	0	7.1	7.1	0	84	90	f	Progressive	Concerto	Concerto	FLEX28	FLEX28	Sonnet	Sonnet
Sbj 4	67	6.55	7.59	1.04	6.55	7.59	1.04	8.54	7.5	1.04	X	X	f	Sudden HL	Concerto	Concerto	FLEX28	FlexSoft	Sonnet	Sonnet
Sbj 5	75	22.98	25.86	2.87	21.98	24.86	2.87	7.17	4.29	2.87	X	X	f	Otosclerosis	Concerto	Synchrony	FLEX28	FLEX28	Sonnet	Sonnet
Sbj 6	59	20.79	15.24	5.56	18.79	13.23	5.56	3.38	8.94	5.56	X	X	f	Progressive sensorineural HL	Synchrony	Sonata	FLEX28	FLEX28	Sonnet	Sonnet
Sbj 7*	63	58.84	59.67	0.84	47.92	48.81	0.89	4.32	3.42	0.89	X	X	f	Congenital sloping HL/Progressive	Synchrony	Synchrony	FLEX24 (EAS)	FLEX28	Sonnet	Sonnet
Sbj 8	52	33.47	33.47	0	31.47	31.47	0	3.88	3.88	0	X	89.5	m	Traumatic brain injury	Concerto	Concerto	FLEX28	FLEX28	Opus 2	Opus 2
Sbj 9	29	22.92	12.79	10.13	12.68	2.55	10.13	5.68	15.81	10.13	90	88	m	Progressive sensorineural HL, congenital	Synchrony	Pulsar	FLEX28	Standard	Opus 2	Opus 2
Sbj 10	61	1.78	2.72	0.94	0.78	1.72	0.94	8.6	7.66	0.94	X	X	m	Meniere’s disease, progressive	Concerto	Concerto	FlexSoft	FLEX28	Opus 2	Opus 2
Sbj 11	55	45.12	46.57	1.45	44.12	45.57	1.45	6.38	4.93	1.45	X	X	f	Progressive sensorineural HL	Concerto	Synchrony	FLEX28	FLEX28	Sonnet	Sonnet
Sbj 12	37	34.03	34.6	0.58	34.03	34.6	0.58	1.46	0.88	0.58	87	83	f	Perilingual HL, progressive	Synchrony	Synchrony 2	FLEX28	FLEX28	Sonnet	Sonnet 2
Sbj 13*	50	42.34	42.97	0.63	38.53	39.16	0.63	7.98	7.35	0.63	87	87	f	Prelingual progressive sensorineural HL	Concerto	Concerto	FLEX28	FLEX28	Sonnet 2	Sonnet 2
Sbj 14	74	51.61	50.35	1.26	44.61	43.35	1.26	7.91	9.17	1.26	X	X	f	Progressive sensorineural HL	Concerto	Concerto	FLEX28	FlexSoft	Opus 2	Opus 2
Sbj 16	91	11.74	16.08	4.34	11.74	16.08	4.34	6.81	2.47	4.34	83	84	f	Progressive sensorineural HL	Concerto	Synchrony	FLEX28	FLEX28	Opus 2	Sonnet
Min	29	1.78	2.72	0	0.78	1.72	0	1.46	0.88	0	83	83	–	–	–	–	–	–	–	–
Max	91	58.84	59.67	10.13	47.92	48.81	10.13	8.6	15.81	10.13	90	90	–	–	–	–	–	–	–	–
Mean	58.31	29.98	29.65	2.28	26.24	25.91	2.28	6.09	6.41	2.28	86.2	86.9	–	–	–	–	–	–	–	–
Median	59	33.47	33.47	1.04	27.96	27.96	1.04	6.81	7.1	1.04	87	87.5	–	–	–	–	–	–	–	–

Demographic data are also provided for sex, etiology, implant, electrode array, and processor type. x: not measured.

*The subjects suffered from auditory dyslalia.

### 2.2. Speech recognition testing (measurement procedure and stimuli)

All measurements were administered in a double-walled sound attenuating audiological booth, which fulfilled the requirements of the standard DIN EN ISO 8253-1 (2011). The stimuli were presented *via* calibrated loudspeaker (JBL Control 1, Harman, Garching, Germany). The subject faced the loudspeaker at a one meter distance. Two speech tests were conducted, the German matrix sentence test (Wagener et al., 1999) and the Freiburg monosyllable test (Hahlbrock, 1953). The measurements were performed separately for the two ears, removing the audio processor from the contralateral side, using the clinically adjusted audio processor configurations (i.e., threshold and most-comfortable loudness levels and compression). The microphone directional sensitivity was set to “omnidirectional” and noise reduction and automatic functions were disabled to create a uniform testing condition between the users.

#### 2.2.1. German matrix sentence test

In this test, the participant was presented with a spoken German sentence in the presence of masking noise. Each sentence consists of five words with the structure “Name-Verb-Number-Adjective-Object.” The sentences are syntactically correct but semantically unpredictable. The participant was then asked to select on a touch screen the words they heard among ten alternatives for each word. The speech material is balanced to represent the phonetic variety of the German language. A stationary noise comprising the long-term average spectrum of the speech material (Wagener et al., 1999) was used as the masking noise. Speech presentation level was fixed at a level of 65 dB SPL. In the beginning of the test, noise was presented at + 5 dB SNR (60 dB SPL) and thereafter the SNR was adjusted automatically to adaptively measure the SNR corresponding to a 50% correct classification rate, reported as the speech reception threshold (SRT). For more general information on international language matrix tests, please refer to the manual (HörTech gGmbH, 2019).

#### 2.2.2. Freiburg monosyllable test

This test consists of 20 lists, each containing 20 monosyllabic words. For each measurement two of the lists were randomly selected. The words were presented to the listeners at 65 dB SPL in quiet. The listeners were instructed to repeat the words. The percent of words repeated correctly was recorded. Test results of the two presented lists were averaged.

### 2.3. Evaluation of the speech band importance function for improving the accuracy of the outcome measure

**FIGURE 2:**
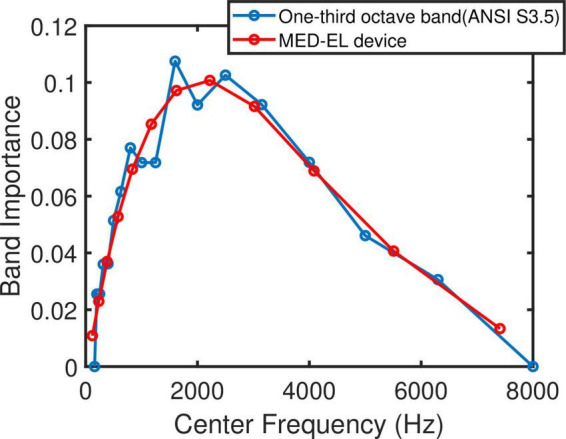
One-third octave band importance function for monosyllables of speech in presence of noise (Supplementary Table 2, ANSI S3.5 1997) in blue and adapted to MED-EL default filter bank setting in red.

The band importance function for one-third octave bands and for monosyllables of speech in noise introduced by ANSI S3.5 (1997- Supplementary Table 2) was adapted to the MED-EL default filter bank setting. The number of bands and their equivalent center frequencies of the one-third octave band differ from the filter bank setting of the MED-EL speech processor. A fourth-degree polynomial was therefore fitted to the above-mentioned band importance function and evaluated at MED-EL default center frequencies. This procedure resulted in 12 weights for the 12 electrodes. Figure 2 shows the original band importance function from ANSI S3.5 and the function adapted to the default center frequencies of MED-EL devices. The IPGE_*slope*_ for each individual electrode was then multiplied with the respective adapted weight for that electrode to reflect the importance of the speech information transmitted *via* that band for speech intelligibility. In cases of deactivated electrodes, the same adapted weights as depicted in Figure 2 were used. For each ear, the across site mean (ASM) of the weighted IPGE_*slope*_ was calculated. These ear-specific weighted IPGE ASMs were then used to investigate the correlation between these measures of cochlear health and the two speech perception measures.

### 2.4. Impedance measurement

Electrode impedances were measured *via* impedance field telemetry (IFT) using the clinical software (MAESTRO 7.0, MED-EL Medical Electronics, Innsbruck, Austria) with the MAX Programming Interface (MED-EL Medical Electronics, Innsbruck, Austria) and a suitable coil. This results in measurements of the implant’s supply voltage, and the impedance values of the 12 implanted electrode contacts. For the purpose of the study, the measurement results were exported (using the scientific export) from the clinical software into XML files, and the supply voltage and the electrode impedance values were extracted for determining the compliance limits. The extracted values were also used in the statistical analysis of the results.

### 2.5. Loudness-based measurements

A custom-made MATLAB-based (The MathWorks, Natick, MA, United States) research software was used to perform loudness-based measurements with pulse-forms and sequences identical to those used in the following experimental measurements. The MATLAB program communicated with the implant using the Research Interface Box 2 (RIB2) Dynamic Link Library (dll), rib2.dll, Version 1.73.0.0, 64 bit (Department of Ion Physics and Applied Physics, University of Innsbruck, Innsbruck, Austria), the MAX Programming Interface and a suitable coil. Threshold (THR) and maximum acceptable level (MAL) stimulation charges were measured using manual control for all active electrodes using cathodic-leading biphasic stimuli with IPG of 2.1 μs, and repeated for an IPG of 30 μs, in sequences of at least 400 ms duration to allow sufficient loudness integration. The phase duration was the same as that selected for the following eCAP measurements. The amplitude could be increased up to the compliance level which was calculated after impedance measurement.

### 2.6. eCAP measurements

A custom-made MATLAB-based tool was used for eCAP measurements considering the THR and MAL values from the loudness fitting tool. The communication with the implant was the same as the software for the loudness-based measurement.

Electrically evoked compound action potentials were measured using the forward masking (FM) artifact reduction approach [Brown et al. (1990), illustrated in Figure 1 of Baudhuin et al. (2016)]. This method exploits the absolute refractory period of SGNs by implementing a double-pulse paradigm with a sufficiently short inter-pulse-interval. Theoretically, the technique results in a voltage trace containing the neural response alone. More details on currently applied artifact reduction techniques have been reviewed by He et al. (2017). FM artifact reduction allows investigation of the auditory nerve response to pulses with a specific initial polarity. In this study, all eCAPs were measured both with anodic-leading (FMA) and with cathodic-leading (FMC) pulses, in order to investigate the polarity-specific behavior of neural responses. For each parameter set, 50 sweeps were recorded and averaged. An AP artifact reduction method was implemented by averaging the FMA and FMC probe responses. It should be noted that FMA and FMC probes were not measured consecutively, which is different to how AP is implemented in clinical software. In all cases, eCAPs were measured using two IPGs, 2.1 and 30 μs. The combination of polarities and IPGs resulted in six different conditions: FMA 2.1, FMA 30, FMC 2.1, FMC 30, (virtual) AP 2.1, and (virtual) AP 30.

ECAP recordings were performed in monopolar configuration at a rate of approximately 60 Hz using the standard stimulation ground of the implant. Recording electrodes were by default set to the next more apical active electrode relative to the stimulating electrode (n−1, where n is the electrode number), except for electrode 1 (the most apical electrode) which had electrode 2 as the default recording electrode. When necessary, the location of each recording electrode could be altered by the investigator to yield a clear eCAP based on visual inspection following an initial test pulse. The recording electrode was neighboring the stimulating electrode in nearly all cases. ECAP recordings were obtained with high temporal resolution (stimulator internal sampling rate 1.2 MHz). The measurement delay was set to 120 μs for stimuli with IPG = 2.1 μs and to 149 μs for stimuli with IPG = 30 μs to compensate for the different durations of the respective biphasic stimuli. Both masker and probe signals were measured with the same recording electrode pair. The masker level was 10% higher than that of the probe, except for the highest amplitude step, for which the masker was set to MAL and the probe was set to 95% of MAL. This procedure led to a smaller increase for the last stimulus step in the AGF and to potentially less effective masking, however, it ensured that MAL would not be exceeded.

The default phase duration was set to 30 μs and the default masker-probe interval to 350 μs, but both parameters could be adjusted by the investigator when necessary. For subjects 7 and 10 the phase duration was increased to 40 and 50 μs, respectively, to record eCAPs with sufficient reliability. Subject 14 had different phase durations in right (30 μs) and left (50 μs) ears. AGFs were recorded on all active electrodes, with 10 amplitude steps between and including threshold and MAL, as well as two sub-threshold measurements. The amplitude increment was equidistant. This resulted in a total of 576 eCAPs for each ear, in the case of 12 clinically active electrodes.

To further reduce the influence of the measurement noise, the recorded eCAPs were filtered with a fifth-order Butterworth lowpass filter having a cut-off frequency of 5 KHz. Zero-phase filtering was applied, which effectively doubled the filter order to ten. To avoid potential tilt of the responses, caused by remaining stimulus artifact, biasing the determination of eCAP characteristics, the filtered eCAPs were detrended. To estimate the trend, a weighted linear least-square analysis was applied to the eCAP. Each eCAP trace was divided into three equally long parts and samples within the first, second and third part were multiplied with weights of 0.1, 0.5, and 1, respectively, to emphasize the tail of the response in the estimation of the trend where the presence of the artifact is more pronounced. This allowed more accurate estimation of the trend. This estimated trend was then subtracted from the eCAP. Finally, in order to eliminate the artifact caused by internal circuitry, the response to the lowest subthreshold current level was subtracted from the detrended eCAP.

### 2.7. AGF slope calculation and IPGE_*slope*_

The extrema of the eCAP amplitude time-course falling into the time intervals of approximately 0.02–0.4 ms and 0.3–0.8 ms were chosen to determine the N1 and P2 peaks, respectively. The earliest extremum of the corresponding time interval was chosen as the N1 peak. For the detection of P2 the extremum with the largest amplitude was selected to allow a consistent definition of the peak in cases of double peaks. The default time intervals were modified if no peak was detected, (in particular in the case of a missing N1). Absence of the N1 peak could be driven by an early response of the neurons occurring during the blanking delay and therefore hidden from the measurement system (Lai and Dillier, 2000). Therefore, in such a case, the N1 peak was defined arbitrarily as the amplitude of the signal at 0.03 ms after stimulus onset. For the selection of the P2 peak, the above-mentioned time interval was expanded toward the onset of the signal to compensate for the delay in the recording and to allow the detection of any peaks that occurred earlier than expected.

Amplitude growth function, i.e., the N1-P2 peak amplitude difference as a function of stimulating current level, were calculated for each electrode, each IPG, each polarity and each artifact reduction approach (FM/AP). An automatic AGF selection was performed in order to only estimate AGFs with adequate reliability. The criteria for AGF selection were based on the maximum eCAP amplitude (the N1-P2 peak-amplitude for highest current level must be larger than 120 μV), impedance of the stimulating electrode (must be lower than 10 kΩ), monotonicity of the AGF and the comparison between the maximal AGF slope and the slope of a line fitted to the first three points of the AGFs (the response to the subthreshold and threshold currents) as an estimation of the artifact. For the last criterion, the slope difference should be larger than 0.5 (μV/ μA) unless the slope of the line fitted to the eCAP amplitude measured with subthreshold and threshold currents was smaller than 0.3 (μV/ μA), indicating a mild level of artifact.

The AGF slopes were estimated according to the window method introduced by Skidmore et al. (2022). The input was converted to charge (nC). ECAP AGFs were resampled at 13 data points to handle the non-uniformly sampled data in the original AGF. Subsequently, first order linear functions were fitted to different subsections of the resampled AGF and the slope of the steepest linear function was determined as the slope of the eCAP AGF. Each subset consisted of four points of the resampled AGF, with overlap of three points between subsequent subsets. Only the data points above the noise floor (set to 20 μV) were considered for the analysis. The final AGF slope was the maximum slope that had been determined using this moving window approach. IPGE_*slope*_ was then obtained by subtracting the AGF slope calculated for IPG 2.1 μs from the AGF slope for IPG 30 μs. Figure 1 depicts two exemplary AGFs obtained with IPG 2.1 μs and IPG 30 μs and their corresponding maximum slope.

### 2.8. Statistical analysis and outliers

All data were analyzed using MATLAB 2020a. Single and multiple linear regressions were employed to investigate the relationship between the IPGE_*slope*_ and speech test outcomes, demographics and electrode impedances. The coefficient of determination (R^2^) was calculated based on the Pearson correlation coefficients in each case. These were reported together with the corresponding level of significance. In the case of multiple linear regression adjusted R^2^ was reported to compensate for the effect of over-fitting caused by the moderate sample size of this study. The adjusted R^2^ was defined as.


(1) $$R_{a\,d\,j}^{2}=1-\left[\frac{\left(1-R^{2}\right)\,\left(n-1\right)}{n-k-1}\right]$$

Where n and k were the sample size and number of independent variables, respectively.

In addition to the standard correlation, a weighted Pearson correlation analysis was also implemented to account for missing data due to rejected AGFs, i.e., the cases that the criteria for an automatic selection of AGF by the algorithm were not satisfied. The correlation coefficient was calculated according to.


(2) $$r_{w}=\frac{\sum_{i=1}^{n}\left[w_{i}\,\left(x_{i}-\bar{x}\right)\,\left(y_{i}-\bar{y}\right)\right]}{\sqrt{\sum_{i=1}^{n}\left(w_{i}\,{\left(x_{i}-\bar{x}\right)}^{2}\right)\,\sum_{i=1}^{n}\left(w_{i}\,{\left(y_{i}-\bar{y}\right)}^{2}\right)}}$$

Where x*_*i*_* and y*_*i*_* were samples of the independent and dependent variables of length *n*, and *x̄* and ȳ were the corresponding mean values. In cases where each sample was an ear (in case of analysis of monaural data), the weight for each sample, w*_*i*_*, was the number of electrodes with accepted AGF for both IPG 2.1 μs and IPG 30 μs divided by the total number of electrodes (12). For the analysis of the ear-differences data, the weight w*_*i*_*, was the average of the weights for each ear of the subject. For example, in case of subject 4 and for FMA condition, an acceptable AGF was obtained for both employed IPGs on 10 and 2 electrodes on the right and left ears, respectively, resulting in weights of 0.8333 and 0.1667 for these ears, respectively. For analyzing ear-differences, a weight of 0.5 (average of 0.8333 and 0.1667) was therefore applied.

In the context of weighted Pearson correlation, to calculate the corresponding level of significance, the test value for the Student’s t-distribution was defined as.


(3) $$t=r_{w}\,\sqrt{\frac{n_{w}-2}{1-r_{w}^{2}}}$$

r*_*w*_* was calculated according to Eq. (2). n*_*w*_* was the effective sample size and was defined as the exponent of the entropy of the weights, with weights being normalized to their summed value.


(4) $$n_{w}=e^{-\sum_{i=1}^{n}w_{i}\,\text{ln}\,\left(w_{i}\right)}$$

The corresponding level of significance (*p*-value) to the adapted *t*-value in Eq. (3) was calculated using MATLAB’s default numerical methods as for the standard *p*-value. The degree of freedom was defined as.


(5) $$d\,f_{w}=n_{w}-2$$

To identify outliers for the variables age, duration of hearing loss until implantation (DHL), hearing aid experience (HAE) and CI experience (CIE), the quartiles outlier test was applied. The coefficients of determination for the ranges 0.0–0.3, 0.3–0.6, and 0.6–1.0 were categorized as weak, moderate and strong, respectively.

## 3. Results

### 3.1. Patient data

Table 1 contains subject demographic data. Subjects 9 and 16 were the youngest and oldest participants of this study. Six subjects had residual hearing. The extent of residual hearing was comparable among this subgroup. Subject 7L was the only case of implantation with a short electrode array (Table 1). This subject had the longest duration of HL, and the CI experience was below the mean value of the group data. No residual hearing was observed for this subject at the time of experiment. Subject 2 was the only subject who showed no ear-difference in any of the investigated demographic data (duration of HL, hearing aid experience and CI experience). This subject was implanted with the same electrode array type and wore the same type of speech processor on both sides. In general, the variation in electrode array type was low among the ears tested. Most of the ears were implanted with a 28 mm long electrode array. There was a small difference in electrode length in a few subjects. The ear-differences in duration of HL, hearing aid experience and CI experience were the same because there was no ear-difference in the onset of HL and hearing aid use in any subject. Therefore, the time of implantation was the only cause of variation in all these three demographic data for the ear-differences.

### 3.2. Speech test outcomes and individual patient factors

**FIGURE 3:**
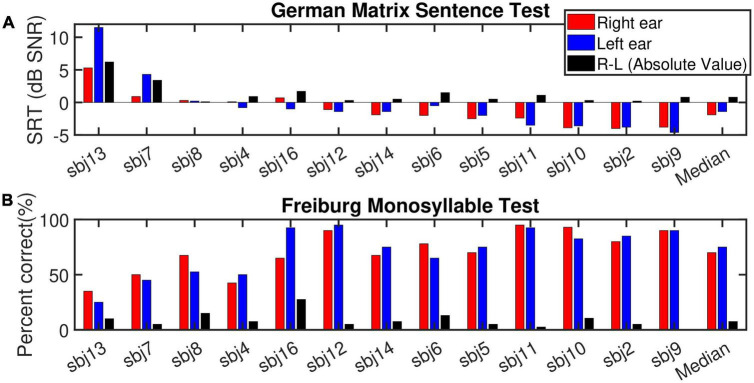
**(A)** Depicts the speech reception thresholds (SRT, dB SNR) measured for the German matrix sentence test for the right ear (red bars), left ear (blue bars) and the absolute value of the ear-differences (right-left, black bars) sorted according to the SRT for the best ear in descending (improvement in speech intelligibility) order. **(B)** Displays the outcome of the Freiburg monosyllable test (% correct). The subjects order and the display are the same as **(A)**.

Figure 3 shows the SRT and percent correct results for the German matrix sentence test and the FMT, respectively (ranked according to the best-ear SRT in a descending order). In general, the SRTs ranged between −5 and 12 dB SNR, with only subject 13 and subject 7L showing SRTs higher than 1 dB SNR. These two subjects were the only ones with (mild) auditory dyslalia. Subject 13 was one of the few subjects of this study who had had a long duration of progressive prelingual HL which probably had a detrimental influence upon speech perception. All these factors have the potential to manifest themselves in the SRT outcome. As subject 13 had notably worse SRT scores in both ears than the other subjects, subject 13 was determined as an outlier and excluded from the analysis related to correlation between speech scores and cochlear health metrics, but was included in the rest of the analysis (with demographic data). Subjects 2, 9, and 10 reached the lowest (best) SRTs. Only subjects 6, 7, 11, 13, and 16 showed ear-differences larger than 1 dB SNR. Given the high measured SRTs for subject 7L and subject 13 (both ears), at least part of the ear-difference in SRT might fall into the test-retest reliability for the matrix sentence test (Hey et al., 2014).

**FIGURE 4:**
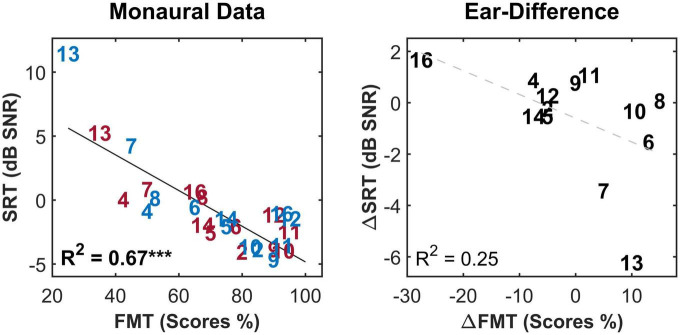
The correlation between the two speech intelligibility measures i.e., speech reception threshold (SRT) and Freiburg monosyllable test scores (FMT scores) for the monaural data **(left panel)** and the ear-differences **(right panel)**. Each subject is represented by a number according to Tables 1, 2. Red, blue and black indicate data from right ear, left and ear-differences, respectively. Black solid lines indicate significant correlations. Dashed gray regression line indicate non-significant correlations. ***p-value ≤ 0.001.

For the FMT, scores ranged between 25 and 95%. No outliers were identified for this test. In the monaural condition, a strong and highly significant correlation (*R*^2^ = 0.67^***^, *p*-val = 0.00, *t*-val = −6.97, df = 24, *R* = −0.81) was observed between the outcomes of the two tests (Figure 4, left panel). Both tests ranked subject 9 and 13 as good and poor performers, respectively. Nevertheless, in case of ear-differences, a comparison of the tests revealed differences in their outcome. A clear difference was apparent for subject 8 who showed the smallest ear-difference for SRT and one of the largest differences for FMT. No correlation was observed between the outcome of the tests for ear-differences (Figure 4, right panel). Furthermore, weak but significant correlations were found between monaural SRTs and demographic data of type age (*R*^2^ = 0.20*) and CI experience (*R*^2^ = 0.21*), data not shown.

### 3.3. IPGE_*slope*_ – Individual electrodes and across-site mean (ASM)

**FIGURE 5:**
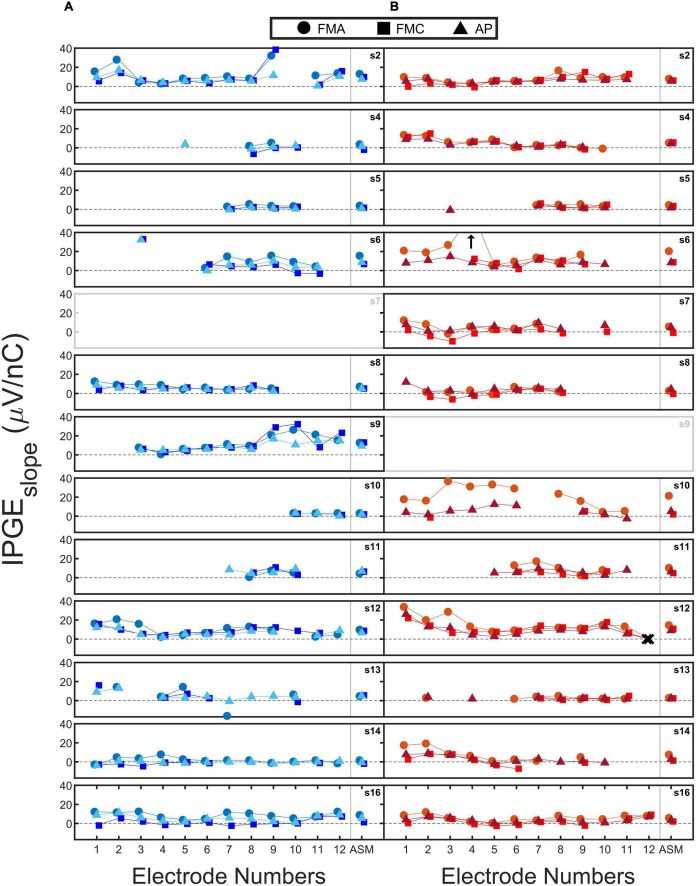
The calculated IPG effect on slope (IPGE_*slope*_) for each of the 12 electrodes (1: most apical and 12: most basal) and their corresponding across site mean (ASM) values. Each subfigure shows the data of one ear. The data from right **(B)** and left **(A)** ears are coded in red and blue. Circles, squares, and triangles mark forward masking with anodic-leading pulse (FMA), forward masking with cathodic-leading pulse (FMC) and alternating polarity (AP) conditions. Crosses indicate the clinically deactivated electrodes. Arrows indicate IPGE_*slope*_ with magnitudes larger than 40 (μV/ nC). For two out of the three conditions for each electrode, the data points are slightly shifted to the left and right to improve visibility.

Figure 5 shows the measured IPGE_*slope*_ for all the subjects. Each subfigure shows the calculated IPGE_*slope*_ for individual electrodes (Electrode 1: the most apical, Electrode 12: the most basal) and their corresponding ASM for one ear. The right and left ears are indicated with red and blue. Circles, squares and triangles mark the three conditions FMA, FMC and AP, respectively. Black crosses indicate clinically deactivated electrodes.

An exemplary case of a successful measurement is subject 16. For subject 16L an acceptable monotonic AGF was obtained for all electrodes and both polarities of the stimulating pulse. Subject 5 is an example for incomplete measurements. For this subject, successful eCAP measurements were obtained for only four electrodes. The calculated IPGE_*slope*_ values for these electrodes were relatively low across the electrodes, possibly indicating poor cochlear health. A clear variation in IPGE_*slope*_ along the cochlea was observed in subject 2L with electrode 2 and 9 (examples of higher IPGE_*slope*_ values) in contrast to electrode 4 and 11 exemplary cases that result in lower IPGE_*slope*_ values. For some of the subjects such as subject 9 a large difference was observed between the right and left ears in terms of the number of electrodes for which IPGE_*slope*_ was available. For subject 9L (who had a congenital component to their HL), successful measurements were obtained for all the three conditions for most of the electrodes. For the right ear of this subject, eCAP measurement was not possible.

For the three conditions further differences were observed between the ears. For example, for subject 4R, the difference in IPGE_*slope*_ between conditions was minor. For subject 10R, however, a difference between polarities was apparent in the estimated IPGE_*slope*_. In the case of FMA and AP, measurements were successful for most of the electrodes, in contrast to FMC which resulted in successful measurements on only 2 electrodes. A noticeable difference was also observed between FMA and AP for the estimated IPGE_*slope*_. Subject 7L and subject 9R were excluded from all analyses due to the extent of the missing data for these ears. Both subjects had a congenital component to their HL. In cases of analyzing ear-differences, data of subject 7 and 9 was excluded for both ears since calculation of ear-differences was not possible.

A comparison of IPGE_*slope*_ measured in this study with the ones measured in a guinea pig model (Figure 7 of Ramekers et al., 2014) showed a smaller magnitude of this cochlear health measure for human subjects. For the 6-week deafened animals in the study of Ramekers et al. (2014) which are comparable to the human subjects of this study in terms of the degree of HL, the measured IPGE_*slope*_ was 6 to 8 (depending on the phase duration) times larger than those measured in this study for AP condition.

### 3.4. Correlation between IPGE_*slope*_ and speech test outcomes

**FIGURE 6:**
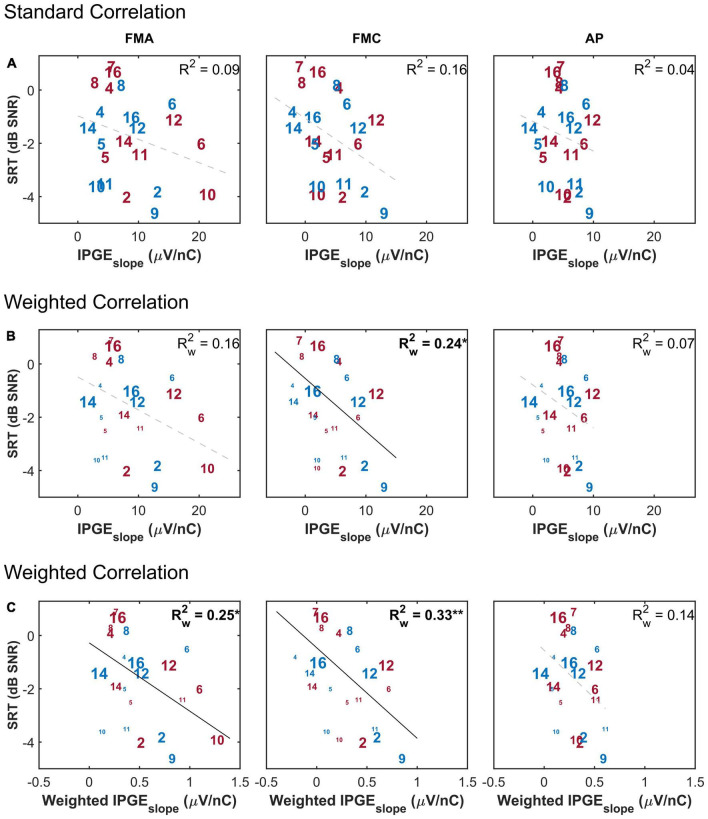
The correlation between speech reception threshold (SRT) and IPGE_*slope*_ ASM for monaural data. **(A)** Shows the result of standard Pearson correlation analysis where each ear contributed equally to the calculated correlation coefficient. **(B)** Depicts the results obtained by applying the weighted Pearson correlation analysis where ears with less missing data contributed more to the calculation of correlation coefficient. **(C)** Shows the result of the weighted Pearson correlation between SRT and weighted IPGE_*slope*_ to account for both missing data and the relative importance of frequency bands for speech intelligibility. The right and left ears are presented in red and blue, respectively. Subject numbers are in accordance to Table 1. The difference in the print size of each number in panels **(B,C)** is proportional to the number of electrodes with successful eCAP AGF measurement for that ear. Black solid lines indicate significant correlations. Dashed gray regression line indicate non-significant correlations. *p-value ≤ 0.05, **p-value ≤ 0.01.

Figure 6 shows a scatter plot of the SRT as a function of IPGE_*slope*_ ASM for the three conditions (FMA, FMC and AP). The upper panel shows the standard Pearson correlation results. In this condition all ears contributed equally to the obtained coefficient of determination, regardless of the extent of missing electrode data. The middle panel shows the weighted Pearson correlation. The size of the number labels representing individual ears was scaled according to the corresponding weight for that ear e.g., the subject 16L has the largest label because, for this ear, eCAPs were measured successfully for all 12 electrodes. In contrast, subject 10L with successful eCAP measurement on only three electrodes has one of the smallest labels.

In the bottom panel, the weighting function was applied to the measured IPGE_*slope*_ on individual electrodes before calculation of ASM of each ear in order to take into account the relative contribution of each electrode’s assigned frequency band to speech intelligibility. Here too, a weighted Pearson correlation was calculated. No correlation was observed between IPGE_*slope*_ and SRT when the effect of missing data was not compensated for and when the relative importance of frequency bands was not taken into account (upper panel). For FMC, a weighted Pearson analysis resulted in a weak but significant correlation (middle panel). In general, for both polarities (FMA and FMC) the highest correlation was observed when the effect of missing data was compensated and the relative importance of frequency bands for speech intelligibility was considered (FMA: $R_{w}^{2}$ = 0.25*, *p*-val = 0.02, *t*-val = −2.45, df = 18.10, *R*_*w*_ = −0.50, FMC: $R_{w}^{2}$ = 0.33^**^, *p*-val = 0.00, *t*-val = −2.95, df = 17.75, *R*_*w*_ = −0.57). For AP, however, this observation resulted in a trend but showed no significant correlation (lower panel).

**FIGURE 7:**
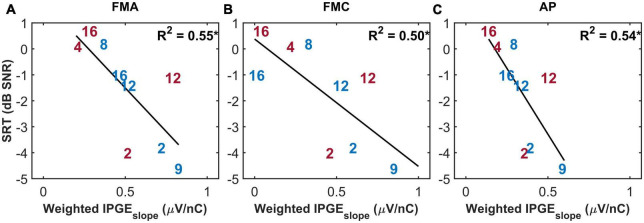
The standard Pearson correlation between speech reception thresholds (SRT) and weighted IPGE_*slope*_ ASM for monaural data. Only the ears with successful eCAP AGF measurement on at least eight electrodes in all the three conditions (**A**: FMA, **B**: FMC, **C**: AP) are included. Right and left ears are plotted in red and blue. *p-value ≤ 0.05.

The purpose of the weighted correlation was to compensate for missing data. Therefore, a comparison was made between the results of the weighted correlation analysis of all ears and a standard correlation analysis of a subset of ears with relatively complete measurements, which we define as AGF for at least 8 out of 12 electrodes in both polarities and for both IPGs (in total, 9 ears). This standard correlation analysis is shown in Figure 7. A stronger IPGE_*slope*_ – SRT correlation was observed for the subset of ears with more successful eCAP measurements in comparison to the result of the weighted correlation analysis of all subjects (Figure 6, lower panel). For the subset of the ears with more successful measurements, the magnitude of the coefficient of determination was comparable between the three conditions (FMA: *R*^2^ = 0.55*, *p*-val = 0.02, *t*-val = −2.92, df = 7, *R* = −0.74, FMC: *R*^2^ = 0.50*, *p*-val = 0.03, *t*-val = −2.66, df = 7, *R* = −0.71, AP: *R*^2^ = 0.54*, *p*-val = 0.02, *t*-val = −2.85, df = 7, *R* = −0.73).

No significant correlation was observed between ear-differences of SRT and ear-differences of IPGE_*slope*_ either for the standard (FMA: *R*^2^ = 0.06, FMC: *R*^2^ = 0.00, AP: *R*^2^ = 0.04) or the weighted (FMA: $R_{w}^{2}$ = 0.06, FMC: $R_{w}^{2}$ = 0.00, AP: $R_{w}^{2}$ = 0.04) Pearson correlation analyses. Applying the weighting to IPGE_*slope*_ to account for the relative importance of each frequency band for speech intelligibility did not result in a significant correlation with SRT (FMA: $R_{w}^{2}$ = 0.06, FMC: $R_{w}^{2}$ = 0.02, AP: $R_{w}^{2}$ = 0.13) when ear-differences were analyzed (data not shown).

**FIGURE 8:**
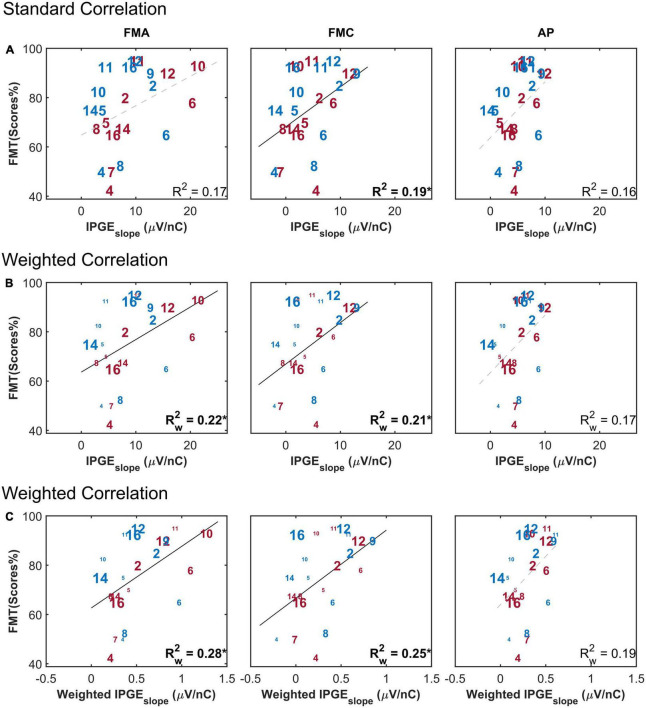
Freiburg monosyllable (FMT) scores as a function of IPGE_*slope*_ ASM for monaural data. The figure has the same structure as Figure 6. **(A)** Shows the result of standard Pearson correlation analysis where each ear contributed equally to the calculated correlation coefficient. **(B)** Depicts the results obtained by applying the weighted Pearson correlation analysis where ears with less missing data contributed more to the calculation of correlation coefficient. **(C)** Shows the result of the weighted Pearson correlation between SRT and weighted IPGE_*slope*_ to account for both missing data and the relative importance of frequency bands for speech intelligibility. The right and left ears are presented in red and blue, respectively. Subject numbers are in accordance to Table 1. The difference in the print size of each number in panels **(B,C)** is proportional to the number of electrodes with successful eCAP AGF measurement for that ear. Black solid lines indicate significant correlations. Dashed gray regression line indicate non-significant correlations. *p-value ≤ 0.05.

Figure 8 depicts the FMT scores as a function of the IPGE_*slope*_ for monaural data and has the same structure as Figure 6. A weak but significant correlation was observed between monaural FMT scores and monaural IPGE_*slope*_ ASM for FMC, as well as for the standard Pearson correlation analysis and in the absence of applying the weighting to account for the relative importance of each band for speech intelligibility. The magnitude of the coefficient of determination was improved when the weighted Pearson correlation analysis was employed. The highest correlation was observed when, in addition to the weighted correlation, the speech-related weighting was also applied, although the correlation was not significant in case of AP (FMA: $R_{w}^{2}$ = 0.28*, *p*-val = 0.01, *t*-val = 2,64, df = 18.10, *R*_*w*_ = 0.52, FMC: $R_{w}^{2}$ = 0.25*, *p*-val = 0.02, *t*-val = 2.46, df = 17.75, *R*_*w*_ = 0.50, AP: $R_{w}^{2}$ = 0.19, *p*-val = 0.05, *t*-val = 2.09, df = 18.67, *R*_*w*_ = 0.43). No significant correlation was obtained between IPGE_*slope*_ ASM and FMT scores either for standard or Pearson correlation or after applying the speech-related weighting when ear-differences were analyzed (FMA: $R_{w}^{2}$ = 0.18, FMC: $R_{w}^{2}$ = 0.01, AP: $R_{w}^{2}$ = 0.04, data not shown).

### 3.5. IPGE_*slope*_ and demographic data

**FIGURE 9:**
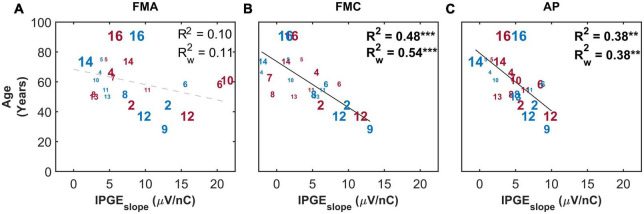
The correlation between IPGE_*slope*_ ASM and age for monaural data. Each column represents one artifact reduction approach (**A**: FMA, **B**: FMC, **C**: AP). Each number represents one ear which is in accordance with Table 1. The right and left ears are plotted in red and blue, respectively. The difference in the print size of each number is proportional to the number of electrodes with successful measurement for that ear. Black solid lines indicate significant correlations. Dashed gray regression line indicate non-significant correlations. **p-value ≤ 0.01, ***p-value ≤ 0.001.

Figure 9 depicts age as a function of IPGE_*slope*_ for the three artifact reduction methods with monaural data. The correlation analysis showed a clear effect of the polarity of the stimulating pulse: a significant correlation was observed only when a cathodic-leading pulse was used for stimulation. The strength of the correlation (*R*^2^ = 0.38, *p*-val = 0.00, *t*-val = −3.71, df = 22, *R* = −0.62) for the AP condition was intermediate between those obtained with FMA and FMC. No correlations were observed between hearing aid experience, duration of HL or CI experience and the IPGE_*slope*_ (data not shown).

**FIGURE 10:**
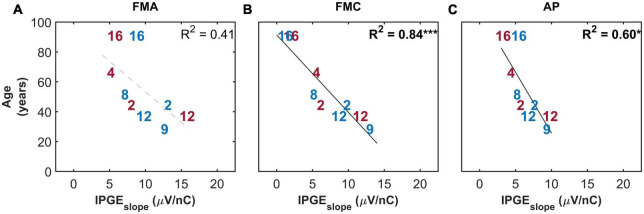
Standard Pearson correlation analysis between age and IPGE_*slope*_ ASM for monaural data. Each column represents one of the three conditions investigated (**A**: FMA, **B**: FMC, **C**: AP). Only the ears with successful eCAP measurement on at least eight electrodes in all the three conditions are included. Right and left ears are plotted in red and blue, respectively. Each number represents one ear which is in accordance with Table 1. Black solid lines indicate significant correlations. Dashed gray regression line indicate non-significant correlations. *p-value ≤ 0.05, ***p-value ≤ 0.001.

Figure 10 shows the correlation between IPGE_*slope*_ and age only for the subset of ears with relatively successful eCAP measurement on at least 8 out of the 12 electrodes. This strict inclusion criterion (applied *post hoc*) strengthened the correlations between the two parameters for all the three artifact reduction approaches. Notably for FMC, a strong and highly significant correlation (*R*^2^ = 0.84, *p*-val = 0.00, *t*-val = −5.98, df = 7, *R* = −0.91) was observed which was considerably higher than the correlations observed in FMA and AP (*R*^2^ = 0.60, *p*-val = 0.01, *t*-val = −3.26, *R* = −0.77) conditions.

### 3.6. Multiple linear regression

A multiple linear regression model was used to investigate the relation of cochlear health measures to demographic data and to electrode impedances. The model was also used to investigate whether considering demographic data and classical impedances in addition to cochlear health, in one model, explains the variation in speech intelligibility to a greater extent. The obtained results for the multiple linear analysis were compared to the standard Pearson correlation in a two-dimensional domain as the reference point. The choice of standard instead of weighted Pearson correlation here was to avoid implementation of weighted multiple regression analysis which requires complex calculations. Two-, three-, four- and five-dimensional models were used. If the dimensionality was higher than two, adjusted coefficients of determination (R_*adj*_^2^) were reported to compensate for overfitting, resulting from an increase in dimensionality. The analysis showed that only age was a significant predictor of IPGE_*slope*_ for FMC and AP conditions. Addition of other investigated demographic factors or electrode impedances did not result in an improvement of model prediction.

Table 2 describes the variations in SRT as a function of the cochlear health measure alone (first row) and together with CI experience. By considering these two variables, more than 50% of the variation in SRT was explained in the case of FMC. Considering both CI experience and IPGE_*slope*_ significantly improved the model prediction in comparison with considering only IPGE_*slope*_ as the independant variable [df(1,19), *F*-val = 15.61^***^, *p*-val = 0.00]. For FMA and AP, the highest explained variation was almost 30%.

**TABLE 2:** Results from the multiple linear regression models to predict the variation in speech reception thresholds (SRTs).

Anodic	B	SE	β	t	*P*	*df*	F	R^2^	R^2^_adj_
**sModel 1**					0.07	(1, 20)	3.62	0.15	
Intercept	−0.72	0.63	–	−1.14	0.27				
IPGE_*slope*_	−1.96	1.03	−0.39	−1.9	0.07				
**Model 2**					**0.01**	(2, 19)	5.57*	0.37	0.3
Intercept	1.16	0.92	–	1.25	0.23				
IPGE_*slope*_	−2.11	0.91	−0.42	−2.31	**0.03**				
CIE	−0.31	0.12	−0.47	−2.55	**0.02**				
**Cathodic**	**B**	**SE**	**β**	**t**	***P***	***df***	**F**	**R^2^**	**R^2^_adj_**
**Model 1**					**0.02**	(1, 20)	6.34*	0.24	
Intercept	−0.94	0.45	–	−2.1	**0.05**				
IPGE_*slope*_	−2.77	1.1	−0.49	−2.52	**0.02**				
**Model 2**					**0.0**	(2, 19)	13.3***	0.58	0.54
Intercept	1.65	0.74	–	2.23	**0.04**				
IPGE_*slope*_	−3.66	0.87	−0.65	−4.22	**0.0**				
CIE	−0.4	0.1	−0.61	−3.95	**0.0**				
**AP**	**B**	**SE**	**β**	**t**	***P***	***df***	**F**	**R^2^**	**R^2^_adj_**
**Model 1**					0.15	(1, 20)	2.29	0.1	
Intercept	−0.88	0.66	–	−1.32	0.20				
IPGE_*slope*_	−2.88	1.9	−0.32	−1.51	0.15				
**Model 2**					**0.01**	(2, 19)	7.01**	0.42	0.36
Intercept	1.92	1.02	–	1.89	0.07				
IPGE_*slope*_	−4.55	1.65	−0.51	−2.77	**0.01**				
CIE	−0.4	0.12	−0.6	−3.26	**0.0**				

In model 1, IPGE_slope_ was used as the independent variable. In model 2, IPGE_slope_ and CI experience were used as independent variables.

Significant P-values are marked in bold.

*p-value ≤ 0.05. **p-value ≤ 0.01. ***p-value ≤ 0.001.

### 3.7. Correlation between IPGE_*offset*_ and speech intelligibility

Brochier et al. (2021) compared different methods used for interpretation of changes in eCAP AGF due to changes in IPG using both computational and animal models. They showed a significant correlation between IPGE on level 50% and SGN density in the animal model. No correlation was observed for IPGE_*slope*_ in the same animal model. They concluded that IPGE_*slope*_ in either the linear or logarithmic domain is vulnerable to non-neural factors such as electrode-to-modiolus distance or impedances of the stimulating and/or recording electrodes. As a solution, for human subjects, the authors proposed the IPGE_*offset*_ which was defined as average offset (in dB re 1 nC) in stimulus amplitude between the linearly growing portions of the eCAP AGFs (obtained with short and long IPGs) expressed on logarithmic input-output axis [Figure 9 in Brochier et al. (2021)].

**FIGURE 11:**
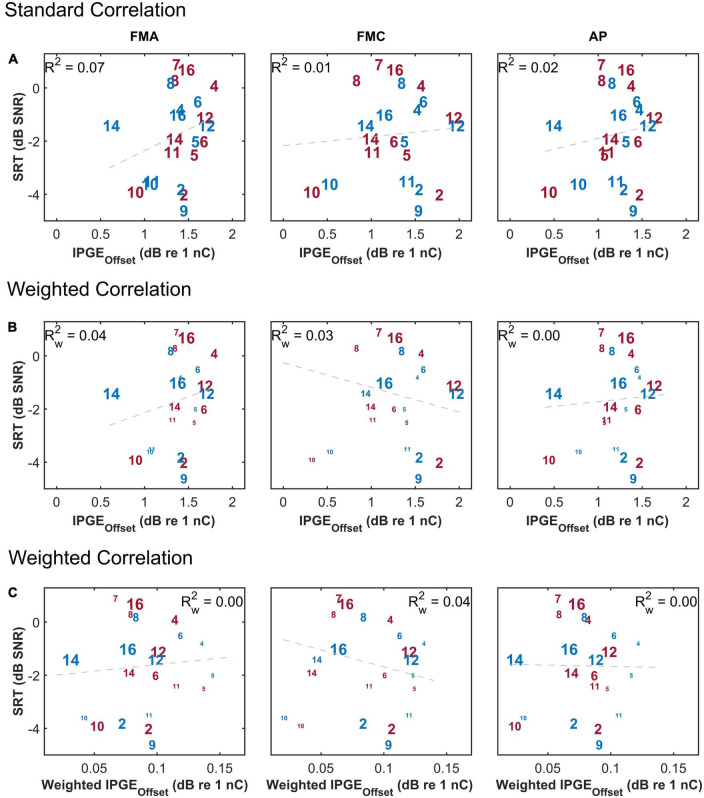
The correlation between speech reception threshold (SRT) and IPGE_offset_ ASM for monaural data. The figure has the same structure as Figure 6.

To compare IPGE_*slope*_ with IPGE_*offset*_, the same analysis introduced by Brochier et al. (2021) was applied to the human data of this study. Two step-sizes for the sampling of N1-P2 amplitudes were used, 0.1 μV as introduced by Brochier et al. (2021) and a step-size of 0.02 μV. Figure 11 shows the results and has the same structure as Figure 6. It depicts SRTs as a function of IPGE_*offset*_ for standard (upper panel) and weighted (middle panel) Pearson correlation and as a function of weighted IPGE_*offset*_ for weighted Pearson correlation (lower panel) to consider both the effect of missing data and relative importance of each frequency band for speech intelligibility. No significant correlation was observed in any case regardless of the employed step-size.

## 4. Discussion

This study investigated the relationship between IPGE_*slope*_, considered to be a measure of SGN survival (Prado-Guitierrez et al., 2006; Ramekers et al., 2014; Schvartz-Leyzac and Pfingst, 2016), and speech recognition in the monaural condition and for ear-differences of each measure. The purpose of analyzing the ear-differences was to provide a controlled experimental condition as reported by Schvartz-Leyzac and Pfingst (2018) by factoring out the interindividual variability in cognitive abilities and other aspects related to processing of the central auditory system in CI users. Furthermore, the influences of stimulus polarity, artifact reduction technique, demographic data and the impact of the application of a weighting function related to the speech band importance function on a measure of cochlear health were all investigated.

### 4.1. Correlation between IPGE_*slope*_ and speech intelligibility and the effect of weighting

For both monaural data and ear-differences, insignificant or very weak correlations were observed between absolute values of IPGE_*slope*_ and speech test outcomes when a standard Pearson correlation was applied, regardless of stimulus polarity. One factor limiting the potential applicability of a correlation analysis was missing data due to unsuccessful eCAP measurements on several electrodes. The extent of missing data was heterogeneous across ears and for both polarities. A weighted Pearson correlation analysis was employed to compensate for the influence of missing data. This resulted in a weak but significant correlation between monaural IPGE_*slope*_ and speech test outcomes for some of the conditions as shown in the middle row of Figures 6, 8.

Another factor influencing the relationship between IPGE_*slope*_ and speech test outcome was the relative importance of each frequency band for speech intelligibility. The importance of speech information is not uniform across the spectrum, but is frequency dependent. Consequently as a result of cochlear tonotopy, degeneration of spiral ganglion cells along Rosenthal’s Canal does not equally impair CI speech outcomes. It is essential to account for this relative importance when relating cochlear health measures to speech intelligibility. This was implemented in this paper by applying the weighting function depicted in Figure 2 to AGF slopes. Employment of this weighting function together with the weighted correlation analysis resulted in an improved significant correlation between IPGE_*slope*_ and speech test outcomes for monaural data and in FMA and FMC conditions. A comparison of the upper rows of Figures 6, 8 with the lower rows reveals the effect of compensation for these two factors. No correlation was observed for ear-differences in any condition.

These findings are in line with those of Imsiecke et al. (2021), who found no correlation between IPGE_*slope*_ and speech intelligibility in CI listeners with residual hearing for the case where the effect of missing data and relative importance of speech information were not considered. In contrast, Schvartz-Leyzac and Pfingst (2018) observed a strong correlation between ear-differences of IPGE_*slope*_ and SRT. The observed difference might partly be due to differences in calculation of IPGE_*slope*_ ASM. In this study, first changes in AGF slopes in response to changes in IPG were calculated for each electrode and subsequently the mean of slope changes for all the electrodes was calculated. In Schvartz-Leyzac and Pfingst (2018), the difference of the means of AGF slopes for single IPGs was reported (most probably to overcome the ambiguities raised by missing data). The two approaches would have been identical in the absence of missing data. In both studies, for some of the electrodes no AGF was obtained at least for one of the IPGs resulting in a difference when changing the order of averaging and subtraction.

Another potential reason behind the contradictory conclusions of the two studies might be the differences in speech understanding scores of the subjects in each study. The ear-differences in SRT for several participants of the study of Schvartz-Leyzac and Pfingst (2018) ranged from a few dB to 10 dB SNR. Whereas in this study, ear-differences higher than 1 dB SNR were measured only for four subjects. Two of these four subjects, subject 7, and subject 13, who showed the largest ear-differences were excluded from IPGE_*slope*_-SRT correlation analysis. The reason behind the exclusion of subject 13 was identification as outlier in terms of speech intelligibility. In case of subject 7, the eCAP measurement of the worse ear was interrupted by the subject, leading to incomplete data. These two subjects were also the only ones who had (mild) auditory dyslalia which is an indication of irregular speech development, possibly leading to a relatively large effect of cognitive processing on performance (Lang-Roth, 2014). Showing evidence for the relationship of IPGE_*slope*_ and SRT ear-differences might have been more straightforward with SRT ear-differences in the range of 10 dB SNR, however, recruitment from the relatively large patient database of our center resulted in no such participants for this study. Consideration of sequential delays between implantation dates on both sides also played a role for subject inclusion. Subjects who received their second implant shortly after the first implantation were prioritized (except for subject 9). A comparison of the ear-differences in CI experience showed a tendency toward larger differences for the study of Schvartz-Leyzac and Pfingst (2018). Differences in speech testing materials used may also contribute to the difference in speech-test outcomes. The tests might not be equal in their semantic content and consequently in engagement of cognition. In terms of SRT measurement, two methodological differences were observed between the two studies. First, Schvartz-Leyzac and Pfingst (2018) employed a step-size of 2 dB to obtain the adaptive track. However, in this study an adaptive step-size was used which was varied depending on the subject’s response and might have enabled a more accurate estimation of the speech reception threshold. Second, Schvartz-Leyzac and Pfingst (2018) kept the level of the mixed signal (speech + noise) constant. In present study, the level of noise was kept constant and the level of speech was varied to obtain the desired SNR. Additionally, differences in electrode array types, test language, and inclusion of two subjects with the history of explantation/reimplantation by Schvartz-Leyzac and Pfingst (2018) may contribute to the differences in the outcome of the two studies.

Standard Pearson correlation analysis of the ears with the most successful eCAP measurements showed mild but significant correlations between IPGE_*slope*_ and SRTs. This result supported the validity of weighted correlation as a method to partly compensate for the effect of missing data. It also supported the assumption that IPGE_*slope*_ would be more suitable for assessing cochlear health if complete eCAP measurement sets were available. The strength of correlation was roughly similar for the three conditions. Based on this result, it could be concluded that, in case of high quality eCAP measurement, polarity might not be influential in correlating cochlear health to speech intelligibility in quiet or in stationary noise. However, an analysis with the same subset of subjects relating age to IPGE_*slope*_ did exhibit differences between the three conditions (Figure 10).

Significant correlations were observed between SRT and FMT scores for monaural listening but not for ear-differences of the two measures. The same pattern was obtained for the analysis of IPGE_*slope*_ and SRTs. The large number of subjects in this cohort with progressive HL as the etiology may suggest a high number of genetic causes, which can be more likely to be symmetric. These findings give rise to doubts about the general applicability of an analysis of ear-differences of speech test results. While it appears feasible in certain groups of listeners with relatively large ear-differences in SRT, such as those reported by Schvartz-Leyzac and Pfingst (2018), transferring the approach of analysis of ear-differences to a random selection of bilateral CI users might not always yield a useful result.

### 4.2. Correlation between IPGE_*offset*_ and speech intelligibility

Brochier et al. (2021) compared different methods used for interpretation of changes in eCAP AGF due to changes in IPG using both computational and animal models. They showed a significant correlation between IPGE on level 50% and SGN density in the animal model. No correlation was observed for IPGE_*slope*_ in the same animal model. They concluded that IPGE_*slope*_ in either the linear or logarithmic domain is vulnerable to non-neural factors such as electrode-to-modiolus distance or impedances of the stimulating and/or recording electrodes. As a solution, for human subjects, the authors proposed the IPGE_*offset*_ which was defined as average offset (in dB re 1 nC) in stimulus amplitude between the linearly growing portions of the eCAP AGFs (obtained with short and long IPGs) expressed on logarithmic input-output axis [Figure 9 in Brochier et al. (2021)].

One potential reason behind the observed difference in the outcome of this study and findings of Brochier et al. (2021) might be due to differences in assessment approaches. Unlike with computational and animal models, the accurate estimation of level 50% is difficult in most of the human subjects. When the AGF is sampled at only 12 current levels, as was done in the present study, a robust estimation of level 50% requires the AGF to reach the inflection point. This was rarely observable in our data and is generally difficult to measure post-operatively with stimulation levels below the loudest applicable presentation level. As a substitution to level 50%, the method based on averaging of current offset for different voltage levels (and not only level 50%) was implemented as suggested by Brochier et al. (2021). This change in the approach for estimation of IPGE_*offset*_ might at least partly explain the difference in the outcome of these two studies. The results obtained in this study are in line with the findings of Kim et al. (2010) who employed a very similar approach for the calculation of IPGE_*offset*_ and reported no significant correlation between this measure and the speech performance in human subjects.

### 4.3. The choice of linear domain

Many studies have investigated the proper unit for analysis of psychophysical and physiological measurements in CI users. McKay (2012) investigated the psychometric probe threshold measured using a forward masking paradigm. The author argued that the ratio or logarithmic units are the best for estimation of probe thresholds because only in these domains are the effects of electrode-neuron distances canceled out, and only the effective change that neurons experience remains. For example, increasing the stimulation current from 100 to 200 μA might result in an increase of 0.5–1 μA in one case and from 1 to 2 μA in other case. In both cases the effective current received by neurons was doubled as a response to doubling the stimulation current, but there is a difference in the raw increment. The author argued that the ratio and logarithmic domain can reflect this effect but not the linear domain.

Brochier et al. (2021) used the same argumentation for the calculation of IPGE_*offset*_ and applied the IPGE_*offset*_ in the logarithmic domain as a cochlear health measure robust against non-neural factors such as variation in electrode-neuron interfaces or variation in the stimulating current level. It should be noted that as IPGE_*offset*_ is a differential measure, a logarithmic transformation not only compensates for different field gradient strengths, i.e., effect current at the recruited population, but also removes any time related effects. Degeneration affects the degree of the temporal integration of neurons. Therefore, a reliable estimation of cochlear health requires a measure which should be sensitive to changes in temporal integration. This argumentation was confirmed with the outcome of this study which showed a significant correlation between IPGE_*slope*_ in the linear domain and speech test outcome but no significant correlation for IPGE_*offset*_ in the logarithmic domain. The observation that the IPGE in the linear domain showed only a significant effect could therefore be explained by differences in how the neural population could integrate over time, and that this information is removed by an analysis in the logarithmic domain.

The findings of this study are in line with the study of Takanen et al. (2022) who modeled three cochlear health measures, (1) IPGE_*slope*_ in the linear domain, (2) relative IPGE_*slope*_ (ratio of slopes), and (3) IPGE_*offset*_ in the logarithmic domain. They investigated the effect of electrode-neuron interfaces and cochlear health (defined as the number of surviving SGN). They reported that only IPGE_*slope*_ in linear domain was sensitive to cochlear health, although it was also affected by variation in electrode-neuron distance. Relative IPGE_*slope*_ and IPGE_*offset*_ in logarithmic domain were not sensitive to either factor.

### 4.4. Analysis of age and other demographic data

Cochlear implant research has already shed light on the relationship between some of the cochlear health measures and demographic data. A correlation between the duration of HL and AGF slope was reported by Schvartz-Leyzac and Pfingst (2016). IPGE on threshold and level 50% were also correlated to duration of HL in the study of Imsiecke et al. (2021). In the present study, a strong correlation was observed between age and IPGE_*slope*_ for FMC but not for FMA (Figures 9, 10). This may highlight a higher diagnostic power of cathodic-leading pulses for a particular aspect of cochlear health. The physiological decrease in human SGN populations (Zimmermann et al., 1995; Otte et al., 2015) with age suggests that a measure sensitive to degenerative processes such as demyelination and loss of SGN PPs as well as subsequent SGN death would therefore exhibit a negative correlation with age. Modeling studies (Rattay, 1999; Rattay et al., 2001a,b; Resnick et al., 2018) elaborated on why cathodic leading pulses are less effective than anodic pulses in eliciting spikes in the region beyond the cell body of SGNs with degenerated PPs. PPs of SGNs degenerate as a consequence of sensorineural deafness (Glueckert et al., 2005) or age-related HL (Kumar et al., 2022) following decreased neurotrophic support from the organ of Corti and from supporting cells (Zilberstein et al., 2012). One finding of this study is that cathodic-leading stimuli are better suited as an electrophysiological marker for SGN degeneration than anodic-leading stimuli. Degenerated PPs should consequently be assessed more reliably with FMC. Observation of a strong negative correlation between age and IPGE_*slope*_ for FMC but not for FMA in this study (Figure 9) suggests that cathodic-leading pulses may in fact be more sensitive for assessment of degeneration of peripheral process and thereby of cochlear health. Previous studies (Jahn and Arenberg, 2019a,b) investigated the relationship between the polarity effect on behavioral thresholds using triphasic stimuli without interphase gap found evidence for increasing cathodic thresholds with increasing age, which are in agreement with the findings of the present study. However, previous electrophysiological investigations have been conducted with similar hypotheses regarding the polarity effect and cochlear health, but generated less conclusive results (Hughes et al., 2017, 2018). The relatively small sample size in such studies is always a statistical obstacle when attempting to generalize and compare results. However, the current investigation differs from the previous studies in various factors, such as the focus on cathodic-leading stimuli (in contrast to anodic minus cathodic, polarity effect), investigation of individual subjects (in contrast to averaging across all or groups of subjects), correlating with age in years, and investigating the IPGE between 30 and 2.1 μs for different polarities (in contrast to comparisons between polarity effect on eCAP threshold, AGF slope or on MCL). One or several of these factors, as well as a different subject selection may be used to argue for the more conclusive results in this study.

Taken together, our findings provide evidence for the initial hypothesis, that IPGE_*slope*_ may be used as an electrophysiological biomarker for cochlear health when measured with cathodic-leading stimuli. Partial degeneration and / or complete loss of SGNs may both play an important role and this differentiation requires further research. Demyelination of the peripheral process will increase membrane capacitance (Rattay et al., 2001b) and along with possible downregulation of ion channel expression (Pan et al., 2016, 2021), the response times of SGNs to extracellular stimulation will increase. Further models investigating demyelination suggested that response thresholds may be largely unaffected, but response timing may change significantly (Resnick et al., 2018). The vulnerable period in which the second phase can still prevent depolarization caused by the first phase to pass threshold (van den Honert and Mortimer, 1979) has been suggested to range between 8.7 and 16 μs (Rubinstein et al., 2001) but may likely extend during years of deafness such that a 30 μs IPG is no longer sufficient to allow spike initiation in neurons close to threshold and at the borders of the excited SGN population. Setting the shorter IPG to 2.1 μs was suited to have samples of the second phase interrupting the vulnerable period, longer IPGs may not be suited for the choice as the shorter IPG. The etiology of HL was progressive in 10 of 13 subjects (77%) in this study, suggesting ongoing degenerative processes as well as the presence of remaining hair cells and PPs. Further research is required for more detailed investigation of the principles underlying preferential polarity sensitivity in CI users.

### 4.5. Multiple linear regression analysis

Although a significant correlation was observed between cochlear health and speech measures, variations in speech perception among CI users are still large. In order to explain the interindividual variability in speech perception of CI users, more than one factor needs to be taken into account. To realize this goal in the present study, a multiple linear regression model was employed and the variation in speech intelligibility was partly explained. IPGE_*slope*_ was used as the main independent variable. Integration of CI experience in the model in addition to IPGE_*slope*_ resulted in the greatest performance of the model. This is most probably because IPGE_*slope*_ was correlated to age and inclusion of age therefore did not provide complementary information to the model. For the case of FMA, the observed correlation was most likely due to CI experience.

It is important to consider the effect of overfitting with high dimensional models, particularly in cases of a smaller sample size as in the one employed here. Adjusted R^2^ values were reported to compensate for the effect of overfitting. Nevertheless, caution should be taken in interpreting the outcome of these models in particular with respect to the data size of 24 ears. Therefore, it is suggested to repeat this analysis with a larger dataset and (if possible) with less missing data. For the multiple regression analysis, it was not possible to include only the ears with relatively complete eCAP measurements. However, based on the higher correlation observed in case of considering only ears with successful measurements on at least 8 electrodes, it can be concluded that employing such a model with a dataset with less missing data might result in an increase in the predictive performance of the model.

Walia et al. (2022) also employed multiple regression models to predict the speech intelligibility of CI users in noise, however, considering different factors to the ones employed in this study. They explained up to 60% of the variation in speech intelligibility by considering electrocochleography (EcochG) and cognition. Complementary to their study that used EcochG as the cochlear health measure, this study employed IPGE_*slope*_. EcochG has the advantage of being measurable in CI candidates prior to implantation and therefore can be used in the process of decision making for implantation. However, since it is not channel specific, it has limited applications to post-implantation. Whereas eCAP based cochlear health measures, as used in this study, have the potential to determine the cochlear health locally, post-implantation and to be employed for individualized fitting. Walia et al. (2022) reported coefficient of determination (R^2^) and not the adjusted coefficient of determination (R^2^_*adj*_), which might have an increased contribution from overfitting.

For future studies additional factors such as deficits in the fitting of CI processors or issues related to rehabilitation measures may be of interest.

### 4.6. The uncontrolled variables and future work

In this study to assess the performance of IPGE_*slope*_ in the estimation of cochlear health (neural status) the correlation with speech intelligibility was selected. The study design controlled for many of the covarying factors affecting speech intelligibility. The variability in electrode array type was kept as low as possible. However, to completely factor out the interindividual variability in reconstruction of cochlear tonotopy, the information about the length of electrode array should be assessed together with the respective insertion angle and cochlear size. To avoid such a bias, this information should be considered in future.

In an attempt to control for the cognitive ability of the subjects, ear-differences in cochlear health measures and speech intelligibility were employed. However, analysis of the ear-differences revealed limitations to this approach. These limitations include the difficulty in recruiting a large enough number of subjects with between-ear SRT differences higher than 1 dB SNR, and the difficulty of obtaining complete eCAP measurements for both ears in some subjects. Therefore, the analysis of monaural data was preferred in the present study. However, this approach came at the expense of losing control over the cognitive ability (which is a highly individual variable and demonstrated in many studies to be related to performance). Therefore, it might be useful for future studies to assess the cognitive ability of the subjects *via* additional testing in order to describe some of the remaining unexplained variation in speech intelligibility of the CI users.

One other uncontrolled factor influencing speech intelligibility is the spread of excitation. Patterns of spread of excitation vary among the CI users and are affected by features of electrode positioning such as distance from the lateral wall, by the electrode impedances, and by the parameters of the stimulating pulse e.g., pulse amplitude. A large spread of excitation results in interaction between neighboring channels and consequently deteriorates the speech cues. Reliable transmission of speech cues requires focused excitation as well as functioning SGNs. Transmission of deteriorated speech cues by functioning SGNs may result in degraded speech intelligibility. Therefore, to assess the influence of cochlear health on speech intelligibility it is important to control for the spread of excitation. However, it might be difficult to find a measure of the spread of excitation that controls for the survival of SGNs (He et al., 2017). Garcia et al. (2021) took an initial step in disentangling the effect of these two inter-related factors and introduced an approach for estimation of spread of excitation while using neural health vectors to control for SGN survival. Measures based on imaging techniques might also be helpful in bypassing these confounding effect (Noble et al., 2013). Considering the variation in the spread of excitation might help to better explain the outcome of this study.

Electrode impedances also might be indicative of the influence of non-neural factors on cochlear health measures. Impedance measures are regularly assessed in clinical visits and could be used to modify an index of cochlear health without additional effort by the clinician. In animal models, impedance measures have been shown to correlate with intrascalar fibrosis (Ramekers et al., 2021) and with ossification (Colesa et al., 2022), while a distinct relationship between impedance and CI speech outcomes cannot be shown (Prenzler et al., 2020). In future, the relationship between electrode impedances and cochlear health should be investigated to assess the impact of electrode impedances on cochlear health measures.

In addition to objective measures of cochlear health, a subjective measure called charge integration efficiency has been introduced by Zhou et al. (2020). Loudness grows more slowly with an increase in pulse phase duration in comparison with pulse amplitude (for the same delivered charge). The dB difference between pulse amplitude and pulse phase duration dynamic range, i.e., the established chronaxie measure, may be used to estimate the extent of neural degeneration. Zhou et al. (2020) correlated the charge integration efficacy with duration of HL, an indirect measure of cochlear health, as well as with speech recognition (Zhou et al., 2021). In comparison with IPGE_*slope*_, charge integration efficacy might be a faster measure of cochlear health as it can be measured psychophysically in a co-operative subject. Nevertheless, its subjective nature might restrict its possible application e.g., for pediatric cases. Further studies are required to compare IPGE_*slope*_ and charge integration efficacy in terms of their accuracy as well as their vulnerability to missing data.

To calculate the relative importance of each band for speech intelligibility, the band importance function introduced in ANSI S3.5 (1997) was adapted to the MED-EL default filter bank setting. In general, the estimation of speech band importance function could be affected by several factors such as the characteristics of the frequency bands (center frequency and bandwidth, compare Supplementary Table 1 to Supplementary Table 2 for ANSI S3.5-1997) or the language (Jin et al., 2016). Also, natural acoustic speech is different in terms of content from the CI-coded speech (Bosen and Chatterjee, 2016). This latter factor is still an active research topic for CI studies. Even for a certain language, variation in speech material results in differences in the estimated band importance function (CID-22 v.s., NU6, Supplementary Table 2, ANSI S3.5-1997). For any application of band importance function, it is desirable to consider as many of these factors as possible to obtain a function which is tailored to that particular application. It is hypothesized that a tailored band importance function together with complete electrophysiological measurement results in a more accurate prediction of speech intelligibility.

The clinical map of some of the subjects might be different than the default map. Various factors determine the suitable fitting map for individual users in terms of the filter bank setting. The presence of low frequency hearing usually results in a change in the filter bank setting because in such a case the listeners are able to hear the low frequencies acoustically and the CI codes a restricted frequency bandwidth. Another influential factor might be the usage of the anatomy-based fitting which aims at preserving the natural frequency-place map. Here, the amount of the change depends on the insertion depth and the position of the electrodes in the cochlea. Electrode deactivation also affect the filter bank setting and consequently the band importance function. Facial nerve stimulation, open or short circuitry are exemplary common reasons for electrode deactivation which results in the frequency redistribution among the remaining electrodes and depends on the number of deactivated electrodes. The extent of the variation from the default map is individual and ranges from a slight change to a moderate one. Employment of band importance functions adapted to individual maps of the CI users is worthwhile to be tested in future.

## 5. Conclusion

This study investigated the applicability of cochlear health measures for prediction of speech perception capabilities in CI users. We focused particularly on investigating the effect of the polarity of the stimulating pulse and the utility of the band importance function. In conclusion, significant correlations were observed between IPGE_*slope*_ and speech perception outcomes, with equal correlation strength for anodic-leading and cathodic-leading pulses, but not for the ear-differences. We found that reliable relationships between the investigated parameters of cochlear health could only be established when the relative importance of each frequency band for speech intelligibility was taken into account. A significant negative correlation was observed between IPGE_*slope*_ and age. In this case, cathodic-leading pulses resulted in a significant and strong correlation, while anodic-leading pulse showed no significant correlation, supporting the hypothesis that cathodic-leading pulses are better suited for detection of degenerated SGN PPs. The higher sensitivity of younger CI users to cathodic-leading may be due to a larger number of excitable PPs in regions closer to the electrode contact where a cathodic stimulus leads to depolarization more effectively. Missing data was particularly detrimental to the analysis. The highest correlations were observed when the effect of missing data was compensated either by implementation of a weighted correlation or when only ears with relatively complete measurements were included into the analysis. For an accurate estimation of cochlear health (neural status), measurements of high quality eCAPs were essential. Stimulation with a cathodic-leading phase might help to improve the estimation of cochlear health. The results of this study together with further information about current spread, which is assumed to be an individual factor and degrades the spectral resolution of the coded speech, have the potential to explain the observed variation in performance achieved by CI users, partly due to variation in the degeneration level of the auditory periphery.

## Data availability statement

The raw data supporting the conclusions of this article will be made available by the authors, without undue reservation.

## Ethics statement

The studies involving human participants were reviewed and approved by the Ethics Committee of the Goethe University Hospital in Frankfurt (ERB number 44/19), and all subjects gave written informed consent. Subjects received an expense allowance for participation in the study. The patients/participants provided their written informed consent to participate in this study.

## Author contributions

LZ wrote the manuscript and analyzed the data. BM analyzed the data and created the figures. HB and UB designed the study and revised and finalized the manuscript. JT reviewed the study design and the manuscript. CG designed the study and revised the manuscript. All authors contributed to the article and approved the submitted version.
