# Supplementary material for: Factors influencing the relationship between cochlear health measures and speech recognition in cochlear implant users
Source: Front Integr Neurosci. 2023 May 12;17:1125712. doi: 10.3389/fnint.2023.1125712 (PMC10213548; doi:10.3389/fnint.2023.1125712)

Supplementary Material

Factors influencing the relationship between cochlear health measures and speech recognition in cochlear implant users

Ladan Zamaninezhad, Berkutay Mert, Heval Benav, Jochen Tillein, Carolyn Garnham, Uwe Baumann^*^

*** Correspondence:** Uwe Baumann: [Uwe.Baumann@kgu.de](mailto:Uwe.Baumann@kgu.de)

# Supplementary Figures and Tables

**Supplementary Table 1:** Across site mean of IPGE_slope_ in µV/nC shown in Fig. 5 (the top row of the double rows) and across site mean of the weighted IPGE_slope_ shown in the bottom row of Fig. 9 (the bottom row of the double rows, shown in italic) to account for the relative importance of each frequency band for speech intelligibility.

|  | **FMA**  **Right Left** | | **FMC**  **Right Left** | | **AP**  **Right Left** | |
| --- | --- | --- | --- | --- | --- | --- |
| **Subject 2** | 8.0588  *0.5151* | 13.1489  *0.7214* | 6.1505  *0.4594* | 9.8121  *0.6015* | 5.7132  *0.3570* | 7.6471  *0.3927* |
| **Subject 4** | 5.2033  *0.2111* | 3.6854  *0.3469* | 5.5514  *0.2198* | -2.1034  *-0.2142* | 4.3693  *0.1926* | 1.4329  *0.0949* |
| **Subject 5** | 4.5041  *0.4116* | 3.8645  *0.3518* | 3.4677  *0.3023* | 1.5951  *0.1361* | 1.6798  *0.1644* | 0.8397  *0.0789* |
| **Subject 6** | 20.3544  *1.0966* | 15.5584  *0.9717* | 8.7053  *0.7161* | 6.8741  *0.4070* | 8.4479  *0.5052* | 8.7939  *0.5260* |
| **Subject 7** | 5.5282  *0.2667* | N. A | -0.9640  *-0.0110* | N. A | 4.6232  *0.2942* | N. A |
| **Subject 8** | 2.7917  *0.2142* | 7.1612  *0.3695* | -0.5561  *0.0495* | 5.1887  *0.3299* | 4.3755  *0.2373* | 5.2301  *0.2927* |
| **Subject 9** | N. A | 12.6913  *0.8259* | N. A | 13.0225  *0.8509* | N. A | 9.3669  *0.5892* |
| **Subject 10** | 21.3897  *1.2734* | 3.1145  *0.1277* | 1.9489  *0.2250* | 1.9528  *0.0975* | 5.0243  *0.3192* | 2.2034  *0.1183* |
| **Subject 11** | 10.2803  *0.9271* | 4.4643  *0.3710* | 4.8264  *0.4175* | 6.3924  *0.5793* | 6.3758  *0.5131* | 6.9804  *0.6091* |
| **Subject 12** | 15.8399  *0.7916* | 9.8056  *0.5175* | 11.6887  *0.6918* | 8.7115  *0.5267* | 9.8373  *0.5050* | 6.7688  *0.3365* |
| **Subject 13** | 2.8275  *0.2172* | 4.6845  *0.0887* | 2.4028  *0.1700* | 5.5223  *0.1926* | 2.4752  *0.1636* | 4.9786  *0.2431* |
| **Subject 14** | 7.6052  *0.2644* | 1.6563  *0.0994* | 1.2986  *-0.0459* | -1.9307  *-0.0695* | 2.7967  *0.0909* | -0.5742  *-0.0359* |
| **Subject 16** | 5.7893  *0.2820* | 8.8099  *0.4648* | 2.0396  *0.0399* | 1.2350  *0.0131* | 3.2748  *0.1390* | 5.4757  *0.2485* |
| **min** | 2.7917  *0.2111* | 1.6563  *0.0887* | -0.9640  *-0.0459* | -2.1034  *-0.2142* | 1.6798 |  |
| **max** | 21.3897  *1.2734* | 15.5584  *0.9717* | 11.6887  *0.7161* | 13.0225  *0.8509* | 9.8373 |  |
| **mean** | 9.1810  *0.5393* | 7.3871  *0.4380* | 3.8800  *0.2695* | 4.6894  *0.2876* | 4.9161 |  |

**Supplementary Figure 1:** AGF Slope_max_ recordings in µV/nC for IPG of 2.1 µs (A) and 30 µs (B) for the left.


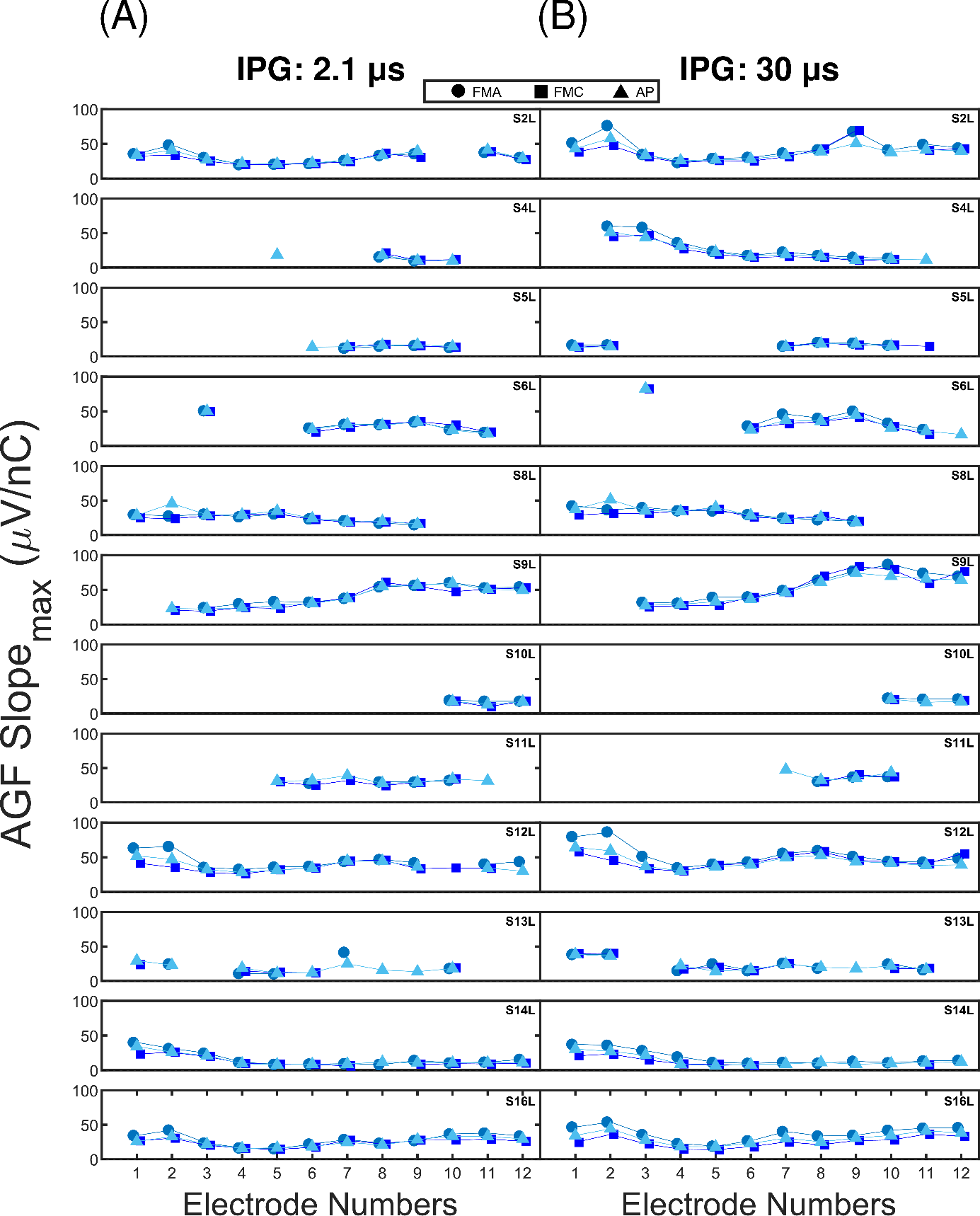


**Supplementary Figure 2:** AGF Slope_max_ recordings in µV/nC for IPG of 2.1 µs (A) and 30 µs (B) for the right.


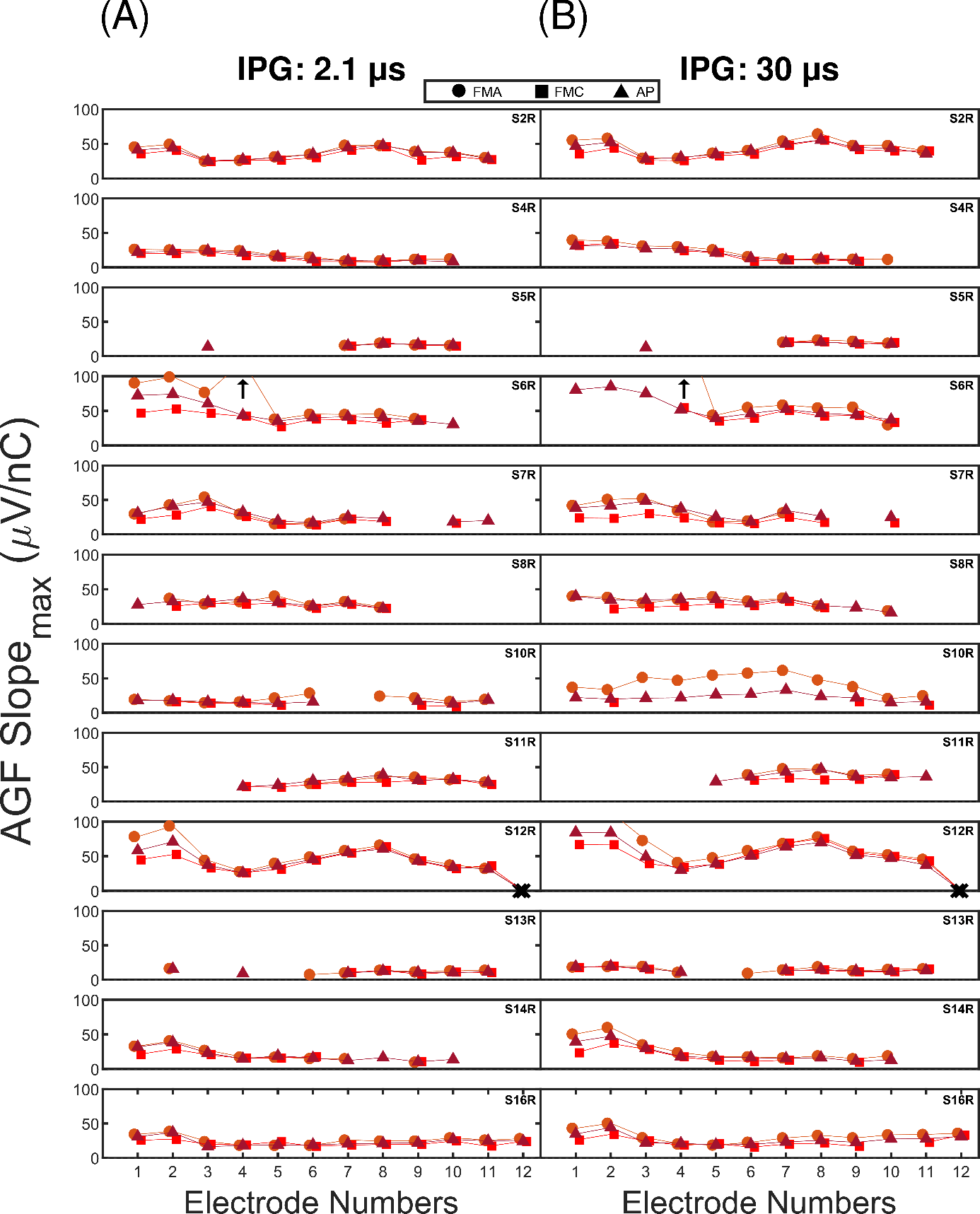

Supplement: Supplementary file 1 [file Data_Sheet_1.docx]
